# Optimization of vitamin K antagonist treatment: Near patient monitoring versus standard of care a parallel group clinical trial in older patients with atrial fibrillation

**DOI:** 10.1007/s00228-025-03878-8

**Published:** 2025-07-12

**Authors:** Margriet van Elp, Maarten Beinema, Jacobus R. B. J. Brouwers, Esther van ’t Riet, Sjef van de Leur, Ildiko Schreiber, Frank G. A. Jansman

**Affiliations:** 1 Department of Clinical Pharmacy, Deventer Hospital, Nico Bolkesteinlaan 75, 7416 SE Deventer, The Netherlands; 2 Department of Cardiovascular Medicine, Anticoagulation Centre, Deventer Hospital, Deventer, The Netherlands; 3 Unit of Pharmacotherapy, -Epidemiology &-Economics, Groningen Research Institute of Pharmacy (GRIP), University of Groningen, Groningen, The Netherlands; 4 Research Centre University Medical Centre Utrecht, Utrecht, The Netherlands; 5 Anticoagulation Clinic, Isala Hospital, Zwolle, The Netherlands

**Keywords:** Coumarins, Acenocoumarol, Phenprocoumon, Frail elderly, Safety, Drug dosage calculations

## Abstract

**Purpose:**

Atrial fibrillation (AF) is common in the elderly population and is associated with a high risk of thromboembolic events. Although non-vitamin K oral anticoagulants (NOACs) are the preferred drugs in antithrombotic therapy for AF, Vitamin K Anticoagulant drug (VKA) treatment is still used in a considerable proportion of patients with AF. Moreover, recent findings revealed that switching VKA to NOAC is associated with more bleeding complications in frail older patients with AF. Standard of care (SOC) monitoring of VKA treatment consists of venous blood sampling and back office dosage advice with a chain of processes and involvement of several health care professionals. We have designed a new procedure for monitoring (Near Patient Therapeutic Monitoring / NPTM) in order to improve the quality and safety of VKA treatment. NPTM consists of INR measurement with a point-of-care (POC) device in the home setting of a patient, performed by one professional and with an instant dosage advice.

**Methods:**

This is a cluster-randomised, parallel group, open label study to compare SOC with NPTM of VKA treatment in patients in a home setting. The follow-up period was one year. The primary outcome was time in therapeutic range (TTR), and secondary outcomes were adverse events (deaths, bleeding and thromboembolic events).

**Results:**

555 Patients were included in the study. After randomisation, 271 patients received SOC and 284 patients received NPTM. The TTR did not differ significantly: 63.71% versus 62.47% (*p* > 0.05) for SOC and NPTM, respectively. Significant differences were found for all-cause death (SOC *n* = 34 versus NPTM *n* = 16, *p* < 0.05, OR 0.47, 95% CI: 0.25–0.87), total number of minor bleedings (79 events in SOC vs 52 in NPTM, *p* < 0.05, OR 64 (95%CI: 0,37–0,81) and all non-major bleedings (100 events in SOC vs 67 in NPTM, *p* < 0.05, OR 0.62 (95% CI: 043–0.90).

**Conclusions:**

NPTM of VKA treatment in AF-patients does not result in an improved TTR when compared to SOC. All-cause death, total number of minor bleedings and all non-major bleedings may be reduced in NPTM, although the study was not powered for these secondary outcomes.

Future studies are needed to determine the cost-effectiveness of NTPM versus SOC.

## Introduction

Atrial fibrillation (AF) is detected in 4% of 60- to 70-year-olds and in 14% of people older than 80 years [1]. Without anticoagulant treatment, patients with AF have a fivefold higher risk for thromboembolic events.

A meta-analysis of 4 trials by Ruff et al. showed a 52% (range: 37–60%) reduction in intracranial haemorrhage for non-vitamin K oral anticoagulants (NOACs) compared to vitamin K antagonists (VKAs) [2]. NOACs are preferred over VKAs, but not in all patients [3]. VKAs are recommended in e.g. patients with mechanical heart valve replacement or moderate to severe mitral stenosis, patients receiving CYP3A4/p-glycoprotein–inducing agents and after bariatric surgery [4]. Also an extreme low (< 50 kg) or a high (> 120 kg) body weight is a factor for considering VKA’s [5]. In addition the FRAIL-AF-study recently showed that switching VKA treatment to a NOAC in frail older patients with AF is associated with more bleeding complications [6]. Unlike VKAs, reversal of life-threatening bleeding under NOAC therapy is still a clinical challenge due to the high costs, availability and limited clinical evidence of specific antidotes for NOACs [7]. Therefore, VKAs are still relevant in anticoagulation therapy for AF.

VKA treatment requires frequent monitoring of the international normalized ratio (INR) due to a narrow therapeutic window. The optimal therapeutic range for AF is an INR range of 2.0–3.0 [8]. Loading and maintenance dose algorithms and pharmacogenetic data can improve the quality of VKA treatment [9]. The Time in Therapeutic Range (TTR), i.e. duration of time in which the patient’s INR values are within the therapeutic range, is a parameter for the quality of the therapy [8].

In the Netherlands, specialized thrombosis centres provide monitoring and guidance for patients with VKAs. In the standard of care (SOC) setting, front-office healthcare workers of a thrombosis centre take blood samples and ask the patient about changes in their actual health status. The back-office staff determines the VKA-dosage for the next period, based on the most recent INR value and the clinical information. These dosages are calculated by a Computerized Decision Support (CDS) program and then communicated to the patient and noted in the medical record.

The SOC requires participation of several professionals, because front-office workers are usually not trained to make dosage adjustments and the back-office staff is not familiar with the clinical aspects and social environment of the patient. As a result, information and treatment errors may occur. Another disadvantage of SOC is the delay in dose-adjustments: the time between a blood sample for INR determination and a new dosage scheme can exceed 24 h with a risk of under- or overtreatment. Self-management of VKAs with point of care coagulometers has been shown to be a very good alternative to SOC [10]. However, in frail elderly individuals this method is often too complicated. Therefore, we developed Near Patient Therapeutic Monitoring (NPTM): a healthcare professional performs INR-controls with a point-of-care (POC) device at a patient’s home and instantly determines the new dosage regime. In this study the safety and effectiveness of NPTM is compared with SOC.

## Materials and methods

### Design

In this open label, cluster-randomised, parallel-group study older AF-patients with VKA treatment were assigned to either SOC (control group) or NPTM (intervention group). All patients were recruited from the Anticoagulation Clinic of the Isala Hospital in Zwolle, the Netherlands. Patients ≥ 65 years with a diagnosis of nonvalvular AF and VKA treatment *(note: only phenprocoumon or acenocoumarol, because warfarin and tecarfarin are not approved in the Netherlands)* in a home care setting were included. Exclusion criteria were: no informed consent, given illiteracy, low communication skills in Dutch language, concerns of the general practitioner, participating in another clinical study and proven non-compliant to medication. Randomisation was based on patients residences: places were divided in regions and an entire region was assigned to either NPTM or SOC. Patients were followed until one year after inclusion (inclusion period: February 2018 to January 2019) or until the study ended (January 2020).

The study was approved by the independent Institutional Review Board Isala Hospital at Zwolle (The Netherlands) and complies with the Dutch Medical Ethical Trial Guidelines.

### Interventions

#### SOC (control group)

**Fig. 1:**
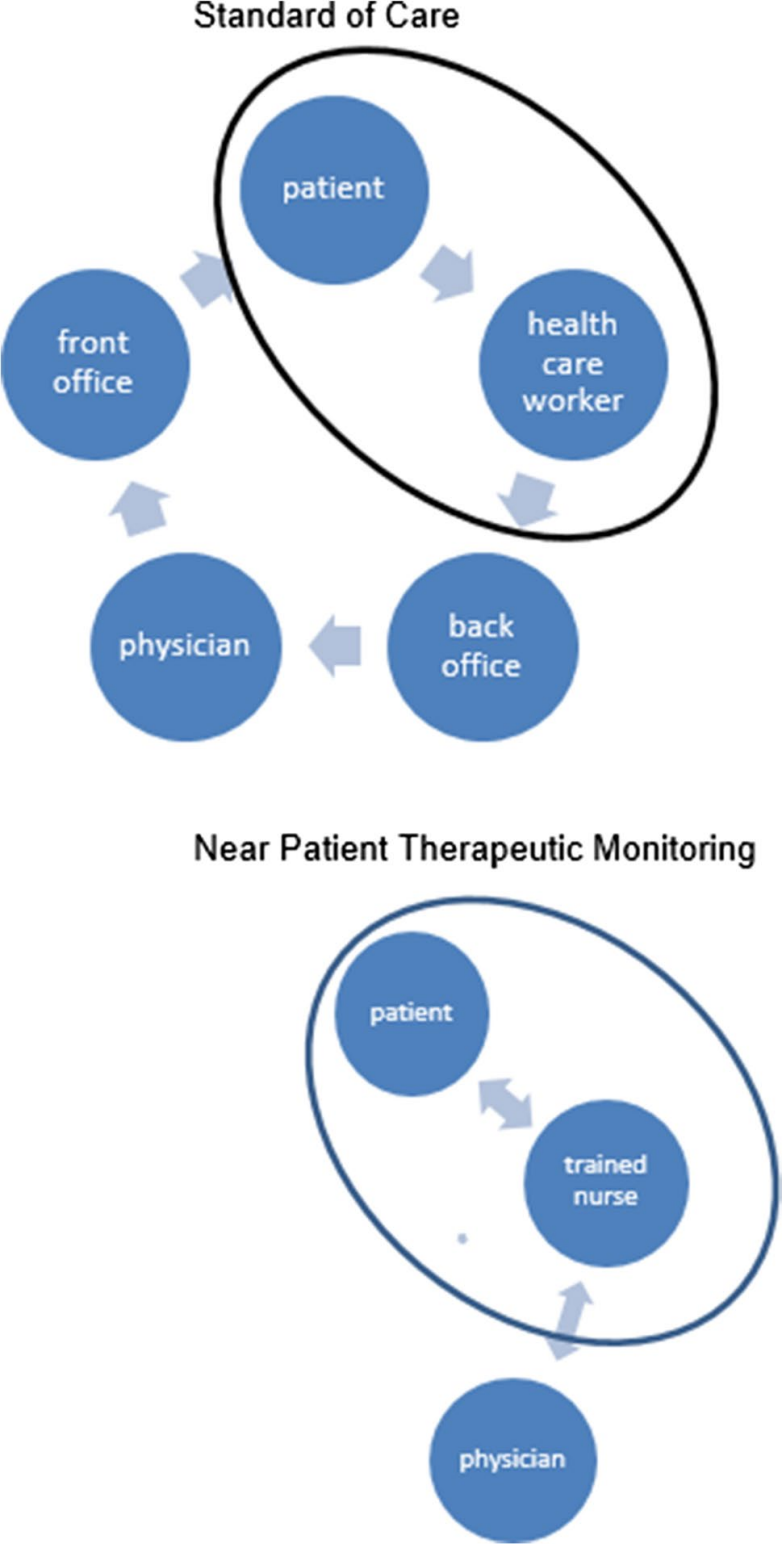
Standard of Care (SOC) versus Near Patient Therapeutic Monitoring (NPTM)

A trained healthcare worker visited the patient at home for INR-measurement with a point-of-care device (POC) and collection of relevant information (e.g. variations in comorbidity and medication use) with a standardized questionnaire. The frequency of this visit depended on the INR-stability, according to the guidelines of the Dutch Federation of Thrombosis Centres. Patient information was noted by back office staff of the Thrombosis Centres in a Web Portal (Centric®-The Netherlands): a patient management system with an integrated, validated dose finding algorithm for VKA-treatment [11]. The dosage scheme for the next period (mostly 1–3 weeks) was calculated by the dose finding system. Medical staff, i.e. qualified nurses or physicians, checked the scheme and made adjustments if necessary. Next, the patient was informed about the new scheme by regular postal delivery, internet and/or (smart)phone (SMS or App) (Fig. 1).

#### NPTM (intervention group)

A trained healthcare worker (nurse) visited the patient at home for INR measurement and information similar to SOC. However, with NPTM, the nurse instantly processed the results and provided a new dosage scheme at the patient’s home. Relevant changes in medical information, mental status and social status were noted in the Web. In case of new comorbidities, contraindications and drug or food interaction, a physician of the back office staff was contacted. The dosage scheme (period 1–3 weeks) was calculated by the same dose finding system as used in SOC. The nurse checked, corrected if necessary and informed the patient. In complex cases, the back office medical staff was consulted. Optionally, the nurse assisted the patient in dispensing the right dosage in medicine dose box.

### Outcome

The primary outcome was the mean TTR of both NPTM and SOC of VKA treatment in nonvalvular AF-patients.

Secondary outcomes were deaths, bleedings and thromboembolic events (any signs of thrombosis or embolism during VKA treatment). Bleedings were defined according to the definitions of the International Society of Thrombosis and Haemostasis. A major bleeding was defined as a fatal bleeding; and/or symptomatic bleeding in a critical area or organ, such as intracranial, intraspinal, intraocular, retroperitoneal, intra‐articular or pericardial, or intramuscular with compartment syndrome; and/or bleeding causing a fall in haemoglobin level of ≥ 20 g/L (1.24 mmol/L) or more, or leading to transfusion of two or more units of whole blood or red cells. A clinically relevant non-major bleeding was defined as bleeding that does not fit the criteria of major bleedings, but where a medical intervention by a healthcare professional was required; and/or hospitalization or increased level of care was needed; and/or a face to face (i.e., not just a telephone or electronic communication) evaluation was needed [12, 13]. Minor bleedings refer to bleedings that do not necessitate medical evaluation.

### Statistics

Based on to the Annual Reports of the Dutch Federation of Thrombosis Centres (FNT), a TTR of 65% is expected for the SOC group (www.FNT.nl). A difference of 5% in TTR between NPTM and SOC is considered as relevant. The mean TTR of all thrombosis centres in the Netherlands is 75% (standard deviation (SD) 20% per patient). At a power of 80% and a 2-sided α-level of 0.05, a minimum of 252 patients is needed in each group. The study was not powered for secondary outcomes, because reliable information in the Annual Report of the FNT was missing.

Age (mean) and sex (%female) of the NPTM and SOC group were compared using an unpaired t-test and chi-square tests respectively. The primary outcome, mean TTR, was calculated by the linear interpolation method of *Rosendaal et. al.* and based on daily measured INR-values [9]. The mean TTR of NTPM and SOC were compared using an unpaired-samples t-test. Odds Ratios (OR) with 95% confidence interval (95%CI) is used for comparison of the secondary outcomes. All analyses were performed by intention-to-treat using Analyse-IT.

### Institutional Ethical Review Board (IRB)

The study was approved by the independent Institutional Review Board Isala Hospital/Zwolle-The Netherlands and is conducted according to the Dutch Medical Ethical Trial Guidelines (www.ccmo.nl).

## Results

A total of 555 patients participated in the study. After randomisation, 271 patients were included in the control group and received usual care (SOC), and 284 patients were included in the intervention group (NPTM). The mean age in the SOC-group was 85.2 years and 73% was female. In the NPTM-group, the mean age was 83,3 years and 65% was female. The CHA2DS2-VASc Score, i.e. the most commonly utilized method to predict thromboembolic risk in atrial fibrillation with regard to risk factors and underlying cardiovascular conditions, of both groups showed no significant difference. Characteristics in both patient groups were comparable (Table 1).

**Table 1:** Patient characteristics

	SOC	NPTM	*p*-value
Number of included patients (n)	271	284	
Mean age (years)	85.2 (SD 5.99)	83.3 (SD 9,47)	0.12 ns
Sex, female (n;%)	199 (73%)	184 (65%)	0.35 ns
Mean CHA2DS2-VASc score	4.0	3.8	0.11 ns
Nr of patients with Score			
1	0	4	
2	20	30	
3	69	82	
4	114	100	
5	41	46	
6	20	14	
7	5	5	
8	1	3	

NPTM: Near-Patient Therapeutic Monitoring; SOC: standard-of-care; Ns: non-significant

CHA2DS2-VASc Score: Congestive heart failure (1 point), Hypertension (1 point), Age (> 65: 1 point, > 75: 2 points), Diabetes (1 point), Previous Stroke/Transient ischemic attack (2 points), Vascular disease (1 point), Sex (female: 1 point)

50 Patients died during the study period: 34 patients in the SOC-group and 16 patients in the NPTM-group. In the SOC-group, 47 patients discontinued the follow-up period for other reasons than death, and 43 patients in the NPTM-group, respectively. Table 2 shows the reasons for discontinuation. Another form of treatment, i.e. non-VKA and non-DOAC, was the main reason of discontinuation in both groups. Furthermore, 12 patients with SOC and 6 patients with NPTM switched to a NOAC during study period.

**Table 2:** Incidence of death by all causes and discontinuation

	SOC	NPTM
Patients (n)	271	284
Patient years (years)	244,6	261,6
Deaths (n)	34	16
Reasons of discontinuation (n)		
Other form of treatment	26	23
Other region	5	1
Dialysis	0	1
NOAC	12	6
Terminal phase	4	12
**Total**	**47**	**43**

### Time in Therapeutic Range (TTR)

No significant difference was found in mean TTR between SOC and NPTM (63,71%; SD 17,23 and 62,47%; SD 17,09; *p* = 0.3951, in the SOC and NPTM-group, respectively).

### Observed events

There were less bleedings observed in patients receiving NPTM. The total number of minor bleedings (79 events in SOC and 52 events in NPTM, OR: 0,55; 95%CI: 0,37–0,81) and non-major bleedings (100 events in SOC and 67 events in NPTM, OR: 0.62; 95% CI: 0.43–0.90) were significant lower in the NPTM-group. Also significant less patients with NPTM died during follow-up period compared to patients with SOC (34 patients with SOC and 16 patients with NPTM, OR: 0.47; 95% CI: 0.25–0.87). There was a non-significant difference between SOC and NPTM in deaths due to an event, major bleedings, clinical relevant non-major bleedings, thromboembolic events and cerebrovascular events (Table 3).

**Table 3:** Observed events in SOC and NPTM

		SOC (*n* = 271)	NPTM (*n* = 284)	Odds ratio	95% CI	p
Death (n)		**34**	**16**	**0.42**	**0.22–0.77**	0.005*
Death due to event (n)		3	1	0,32	0,03–3,05	0,367 ns
Major bleeding (n)		5	1	0.19	0,02–1.62	0,131 ns
Clinically relevant non-major bleeding	Patients (n)	17	14	0,77	0,37–1.60	0,486 ns
	Events (n)	21	15	0,66	0,33–1,32	0.240 ns
Minor bleeding	Patients (n)	55	40	0,64	0,41–1.01	0,053 ns
	Events (n)	**79**	**52**	**0.55**	**0,37–0,81**	0,003*
All non-major bleedings (n)		**100**	**67**	**0,53**	**0,37–0,73**	0,001*
Thromboembolic event (n)		3	2	0,64	0,11–3.82	0,620 ns
Cerebrovascular event (n)		6	2	0,31	0,06–1.56	0,157 ns

## Discussion

We found no significant difference in mean TTR in patients receiving SOC and patients receiving NPTM, in other words: the quality of both therapies does not differ significantly. The TTR in both groups (63,71% and 62,47% in SOC and NPTM, respectively) was lower than the mean TTR (75%) of all thrombosis centres in The Netherlands. However, both groups consisted of older patients with more comorbidities than the standard thrombosis centre population. The TTR values of our study population were therefore expected to be lower in comparison to the standard population.

However the mean TTR did not differ significantly, less bleedings and deaths occurred in the NPTM-group in comparison to the SOC-group. A 38% and 53% reduction of non-major bleedings and deaths, respectively, was found. These results are in line with a Dutch study, the ‘All-In Trial’. *Van den Dries *et al*.* found a 45% reduction in all-cause mortality in integrated care for elderly AF patients when compared with usual care. The integrated care consisted of quarterly AF check-ups by trained nurses in primary care, monitoring of anticoagulation therapy in primary care, and easy-access availability of consultations from cardiologists and anticoagulation clinics [14]. Another Dutch study investigated the effect of integrated care in secondary or tertiary care for patients with atrial fibrillation: nurse-led care vs. usual care by a cardiologist. A 35% reduction of the risk of the composite outcome of cardiovascular hospitalization and cardiovascular death was found in AF-patients receiving nurse-led care. They concluded that nurse-led care of patients with AF is superior to usual care provided by a cardiologist [15]. However, comparison of our study results with the results of the two studies must proceed with caution, since integrated AF care comprises more than management of OAC treatment.

Besides fewer bleeding and death, there are other advantages of NPTM. In SOC there is a delay between measuring and dosing and the back-office worker who determines the dosage does not know the patient. NPTM combines measuring and dosing in one appointment resulting in more knowledge of the patients situation when dosing. In addition, the improved level of communication between the nurse and the patient in NPTM, should lead to a better registration of events. The significant reduction of non-major bleeding in the NPTM-group may have attributed to the improved communication between patient and professional.

Moreover, NPTM is potentially more cost-effective, because the number and total time of professionals involved during one monitoring round is lower compared to SOC. Also, the lower incidence of bleeding events in the NPTM group is theoretically associated with lower costs. Further research is needed to prove cost-effectiveness of NPTM.

We also noticed that more patients in the SOC-group switched to a NOAC in comparison to the NPTM-group. In the NPTM group more patients discontinued therapy when they were in a terminal stage of life. The decision to switch or stop medication was made by the primary physician or the medical doctor in the hospital. In the NMPT group, consultation was more intense between the nurse and the doctor, which may have influenced the decision to stop.

A limitation of our study is a potential selection bias, due to the cluster-randomised design with interventions delivered on an individual patient level. Also the open label design of the study may be a limitation. However, a double-blind controlled trial in this setting is expensive and impractical to realize. In addition, all patients were recruited from a single centre and only phenprocoumon or acenocoumarol were used, which may limit the generalizability of the study results. Finally, the power of the study was not calculated for all secondary outcomes, although there were found significant differences.

## Conclusion

The quality of VKA treatment, i.e. TTR, in elderly AF-patients did not improve in NPTM when compared to SOC. Although the study was not powered for secondary outcomes, NPTM may have influenced bleeding events and deaths: all-cause death, total number of minor bleedings and all non-major bleedings occurred less in NPTM. More research is needed to determine the cost-effectiveness of NTPM versus SOC.

## Data Availability

No datasets were generated or analysed during the current study.
